# A pH-Sensitive Glutathione Responsive Small-Molecule Probe TZ2 Sensitizes Lung Cancer Cells to Chemotherapy by Targeting Tumor Microenvironment

**DOI:** 10.3390/molecules30153081

**Published:** 2025-07-23

**Authors:** Changle Zhong, Minghan Lu, Guanhao Pan, Xintong You, Yan Peng, Shulan Zeng, Guohai Zhang

**Affiliations:** Key Laboratory for Chemistry and Molecular Engineering of Medicinal Resources (Ministry of Education of China), Guangxi Key Laboratory of Chemistry and Molecular Engineering of Medicinal Resources, School of Chemistry and Pharmaceutical Sciences, Guangxi Normal University, Guilin 541004, China; 2022010365@gxnu.edu.cn (C.Z.); 2022010356@gxnu.edu.cn (M.L.); 3033542093@gxnu.edu.cn (G.P.); 18851876191@163.com (X.Y.); pegnyan630@gxnu.edu.cn (Y.P.)

**Keywords:** tumor microenvironment, pH/GSH dual-responsive characteristics, drug resistance

## Abstract

The tumor microenvironment plays an important role in tumor incidence, metastasis, and chemotherapy resistance. Novel therapeutic strategies targeting the tumor microenvironment have become a research focus in the field of biomedicine. In this study, we developed a smart small-molecule probe, **TZ2**, featuring pH/GSH dual-responsive characteristics. **TZ2** exhibits a unique pH-dependent reaction mechanism: GSH is preferentially covalently modified with maleimide groups in acidic microenvironments (pH < 7), while specifically activating nucleophilic substitutions under alkaline conditions (pH > 7). It is worth noting that **TZ2** effectively eliminates intracellular glutathione (GSH) in a time and concentration-dependent manner, demonstrating significant GSH depletion ability in various tumor cell lines. Pharmacodynamic studies have shown that **TZ2** not only inhibits the cell cycle by regulating the expression of cell cycle-related proteins, but also effectively suppresses the cloning ability of cancer cells. Furthermore, **TZ2** significantly increases the sensitivity of drug-resistant cancer cells to cisplatin. By integrating microenvironment modulation, real-time monitoring, and synergistic therapy, **TZ2** provides a novel molecular tool and theoretical basis for tumor theranostics integration.

## 1. Introduction

In recent years, the pathological characteristics and regulatory strategies of tumor microenvironment (TME) have become the forefront direction of tumor diagnosis and treatment [1,2,3]. As a typical feature of TME, abnormal acidification (pH 6.2–6.9) arises from lactate accumulation caused by the Warburg effect in cancer cells [4,5]. This acidic microenvironment not only induces tumor cell dormancy and enhances resistance mechanisms such as anti-apoptosis and autophagy activation but also establishes an immune evasion barrier by suppressing immune cell activity. However, cancer cells are able to proliferate when their interiors remain in a favorable alkaline pH zone [6,7]. Studies indicate that pH imbalance in the TME not only regulates tumor progression but also directly impacts therapeutic efficacy, including chemotherapy drug penetration and radiotherapy sensitivity [8,9,10]. Therefore, precise characterization of the spatiotemporal pH distribution and its molecular mechanisms in the TME holds significant importance for developing novel diagnostic and therapeutic strategies.

External acidification and internal alkalinization of the microenvironment are prevalent in malignant tumors. For TME pH monitoring, existing technologies include electrochemical sensing, ion-sensitive field-effect transistors, colorimetry, and fluorescence spectroscopy [11,12]. Among these, fluorescence imaging stands out as a core tool for in vivo microenvironment monitoring due to its subcellular-level spatial resolution, millisecond-level dynamic response, and non-invasive nature [12]. Small-molecule fluorescent probes, with their high selectivity, sensitivity, and rapid response [13,14,15,16], demonstrate unique value in tracking organelle dynamics. Numerous high-performance pH-responsive probes have been developed and successfully applied in solution systems, live-cell imaging, and even in vivo applications [17,18,19,20,21,22]. However, most existing probes are limited to acidic (pH 4.0–6.0) or near-neutral (pH 6.8–7.4) ranges [23], while probes responsive to neutral-to-alkaline microenvironments (pH 7.0–9.0) remain scarce [24,25]. Some tumor cells have an alkaline environment, and small-molecule drugs cannot be delivered to the target to work when they enter the cell. This technical limitation significantly restricts their application in special tumor models such as hepatocellular carcinoma (HCC, TME pH 7.2–7.6). Therefore, overcoming this bottleneck by developing wide-spectrum pH-responsive smart probes has become an urgent challenge in the field.

Tumor cells have significantly elevated levels of glutathione, which is critical for their survival, proliferation, invasion, and metastasis. Tumor cells have an abnormally active metabolism and produce large amounts of reactive oxygen species (ROS). High levels of GSH, the main intracellular antioxidant, can effectively scavenge ROS and protect tumor cells from DNA breakage, protein inactivation, and lipid peroxidation caused by oxidative damage. Glutathione is also closely related to chemotherapy resistance in tumor cells. Glutathione S-transferase (GST) catalyzes the binding of GSH to a variety of hydrophobic, electrophilic exogenous and endogenous toxins to form GSH-drug complexes (conjugates) with higher water solubility. Therefore, glutathione (GSH), as another critical regulator in the TME, exhibits concentration gradients that provide unique insights for targeted drug design [26]. By constructing GSH-responsive drug delivery systems, tumor cell redox homeostasis can be precisely modulated to disrupt ROS scavenging mechanisms and reverse chemoresistance [27,28]. Based on this fundamental principle, this study proposes an innovative “theranostic integration” strategy: designing dual-responsive molecules capable of dynamic TME sensing and adaptive therapy. These probes would trigger conformational changes in response to microenvironmental cues during resistance development, enabling simultaneous resistance early warning and therapeutic mode switching.

To achieve this goal, we designed probe **TZ2** using naphthalimide as the molecular scaffold [29,30], incorporating pH/GSH dual-responsive modules: a maleimide unit was used for pH-directed covalent modifications, and carbon–sulfur bonds were used to trigger nucleophilic substitution reactions of GSH [31]. This study investigates the sensitivity of **TZ2** to pH and its response ability and mode to GSH, and elucidates its ability to sensitize lung cancer cells to chemotherapy.

## 2. Results

### 2.1. Design and Synthesis of ***TZ2***

To develop a novel therapeutic strategy for the tumor microenvironment, we employed a precise molecular design to construct a smart small molecule probe, **TZ2**, with pH/GSH dual-responsive characteristics. **TZ2** has a naphthalene dicarboximide fluorophore as the electron-donor skeleton, and its innovative structure consists of two key modules: the maleimide unit and the dynamic carbon–sulfur bond.

**Scheme 1:**
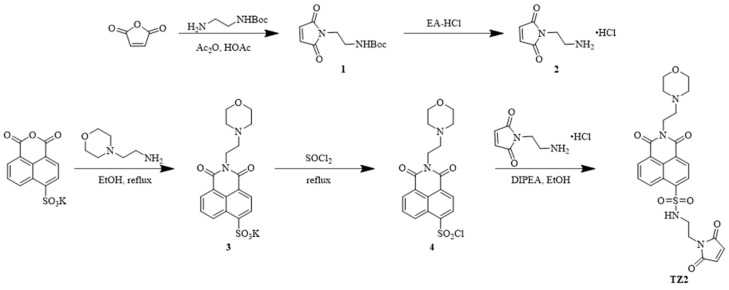
Synthetic route of compound **TZ2**.

An esterification reaction between N-tert-butoxycarbonyl ethylenediamine and maleic anhydride introduced the pH-sensitive component, followed by TFA-mediated deprotection to yield N-(2-aminoethyl) maleimide hydrochloride. Potassium 4-sulfonato-1,8-naphthalic anhydride underwent aminolysis with N,N-diisopropylethylamine under ethanol reflux conditions to produce the key intermediate. A sulfonyl chloride-mediated condensation reaction precisely coupled the naphthalimide fluorophore with the maleimide module, successfully yielding the target compound **TZ2** (Scheme 1).

### 2.2. ***TZ2*** Responses to GSH in a pH-Dependent Manner

**Figure 1:**
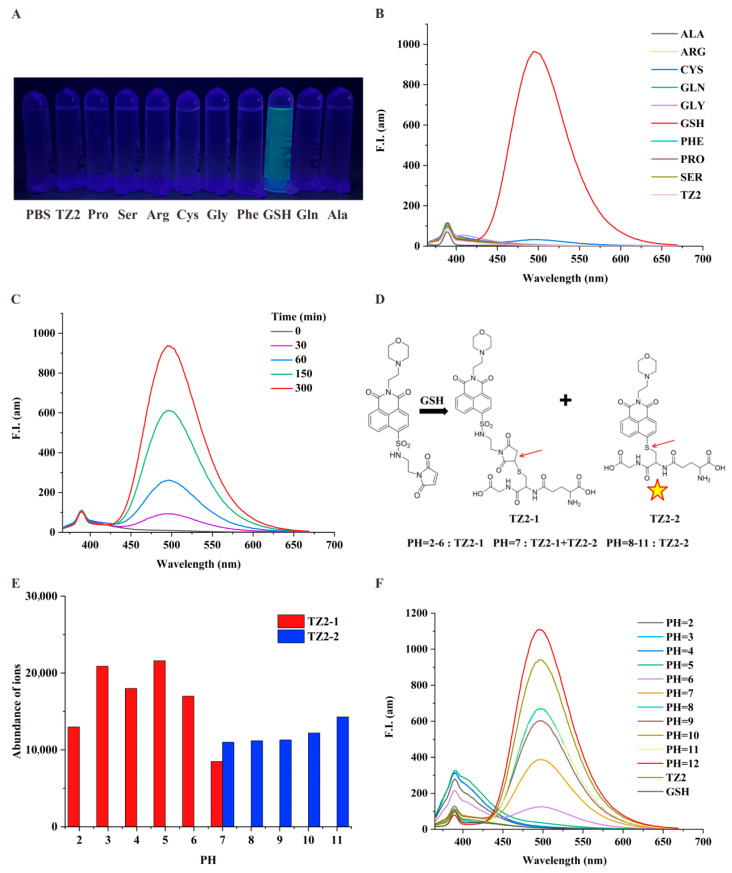
**TZ2** is a pH/GSH dual-responsive small-molecule probe. (**A**) Fluorescence emission of **TZ2** (5 μM) after reacting with various amino acids (100 μM) under a portable UV lamp. (**B**) Fluorescence spectra of **TZ2** (5 μM) after reaction with various amino acids (100 μM). (**C**) Fluorescence spectra of **TZ2** (5 μM) after reaction with GSH (10 mM) for different times (0, 30, 60, 150, 300 min). (**D**) Products of the reaction between **TZ2** and GSH at different pH: **TZ2**-1 and **TZ2**-2 (The arrows represent reaction binding sites and the pentagrams represent the ability of **TZ2**-2 to fluoresce). (**E**) Concentrations of **TZ2**-1 and **TZ2**-2 produced by the reaction between **TZ2** (5 μM) and GSH at different pH levels. (**F**) Fluorescence spectra of **TZ2** after 8 h of reaction with GSH at different pH conditions.

To investigate the specific response of the small molecule probe **TZ2** to GSH [32], we tested the fluorescence change of **TZ2** with a variety of active substances in the biological environment. Results showed that GSH stimulated the fluorescence of **TZ2**, and other thiodiacetyl-containing analytes (e.g., Cys) barely reacted with **TZ2**, confirming the efficient selectivity of **TZ2** for GSH (Figure 1A). Co-incubation of **TZ2** with GSH significantly enhanced fluorescence (Figure 1B). GSH activated the fluorescence reaction of **TZ2** in a time-dependent manner (Figure 1C), indicating that the reaction between the two was slow and continuous, which can achieve the goal of continuous monitoring of the tumor microenvironment.

**TZ2** incorporates a 1,8-naphthalimide fluorophore and a morpholine-based pH-sensing moiety. LC-MS analysis Supplementary Figures S7–S17 under varying pH conditions revealed pH-dependent complex formation (Figure 1D). pH 2–6: Exclusive formation of **TZ2-1** (non-fluorescent); pH 7: Coexistence of **TZ2-1** and fluorescent **TZ2-2**; pH 8–11: Exclusive formation of **TZ2-2** (fluorescent). Fluorescence spectra demonstrated that **TZ2-2** exclusively contributes to emission (Figure 1E). Notably, the fluorescence intensity of the fluorescence reaction showed a good correlation with pH when the pH was greater than 6, peaking at pH 12 (Figure 1F). This biphasic response correlates with **TZ2**′s structural transitions mediated by protonation/deprotonation equilibria at physiological pH ranges.

### 2.3. In Situ Detection of Intracellular GSH Using ***TZ2***

**Figure 2:**
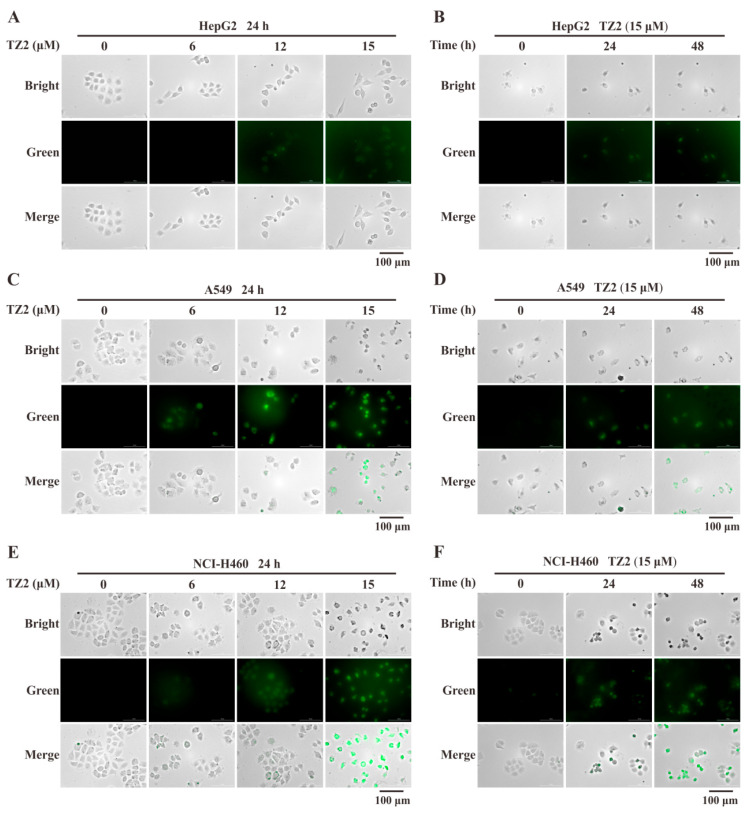
Fluorescence imaging analysis of **TZ2** and endogenous GSH reactions in tumor cells. (**A**,**C**,**E**) Fluorescence imaging of HepG2 (**A**), A549 (**C**), NCI-H460 (**E**) cells treated with different concentrations of **TZ2** (0, 6, 12, 15 μM) for 24 h. (**B**,**D**,**F**) Fluorescence imaging of HepG2 (**B**), A549 (**D**), NCI-H460 (**F**) cells treated with 15 μM **TZ2** (0, 24, 48 h) over time. Data is expressed as mean ± SD (*n* = 3).

To further verify whether the response of **TZ2** to GSH remains in effect in a cellular context. Live-cell imaging analysis was adopted to test the in situ detection ability of **TZ2** for GSH. The results showed that **TZ2** could effectively penetrate cells and react with endogenous GSH to generate fluorescent molecules. **TZ2** was co-incubated with HepG2, A549, and NCI-H460 cells for 24 h, and the **TZ2**-GSH complexes formed in tumor cell lines in a concentration-dependent manner (Figure 2A,C,E). After treatment of all three cell lines with 15 μM **TZ2**, the results showed a time-dependent formation of the **TZ2**-GSH complex (Figure 2B,D,E).

### 2.4. Antitumor Activity of ***TZ2***

**Figure 3:**
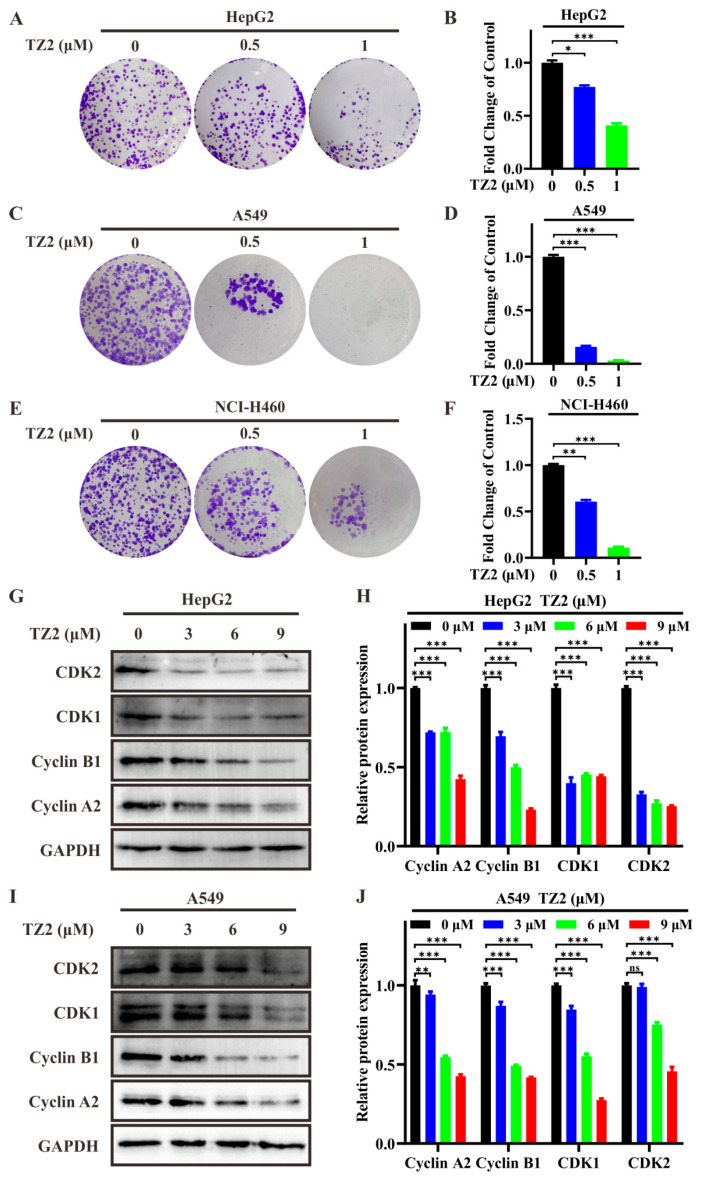
**TZ2** exhibits antitumor activity. (**A**,**C**,**E**) Effect of different concentrations of **TZ2** (0, 0.5, 1 μM) on colony formation in HepG2 (**A**), A549 (**C**), and NCI-H460 (**E**) cells. (**B**,**D**,**F**) Quantitative analysis of the colony formation experiment results in HepG2 (**B**), A549 (**D**), NCI-H460 (**F**) cells using Image J. (**G**–**J**) After treating HepG2 (**G**), A549 (**I**) cells with **TZ2** (0, 3, 6, or 9 μM) for 48 h, cell cycle-related protein expression was detected by Western blot, and the results were quantified using Fiji ImageJ 2025 (**H**,**J**). Columns, means (*n* = 3); bars, standard deviation (^ns^ *p* < 0.05, * *p* < 0.05, ** *p* < 0.01, *** *p* < 0.001).

Dose-response assays were performed to calculate the half-maximal inhibitory concentration (IC_50_) of **TZ2** against various cancer cell lines. The results showed that the IC_50_ value of **TZ2** against A549 cells was 14.51 ± 0.76 μM, whereas the IC_50_ values against NCI-H460 and HepG2 cells were more than 20 μM (Table 1), and it has weak cytotoxicity on normal cell lines WI-38, which indicated that **TZ2** exhibited certain antitumor activity against A549 cells. The antitumor activity of **TZ2** was further assessed by clone formation assay, which showed that **TZ2** was able to inhibit colony formation of different tumor cells (Figure 3A–F). To elucidate the molecular mechanism of the antiproliferative effect of **TZ2**, we evaluated the effect of **TZ2** on the expression of cell cycle-related proteins. The results showed that **TZ2** was able to downregulate the expression of CDK1/2, Cyclin B1, and Cyclin A2 in HepG2 and NCI-H460 cells (Figure 3G–J). These findings indicated that **TZ2** induced cell cycle arrest at the G2/M phase by downregulating the CyclinB1–CDK1 and CyclinA2–CDK2 complexes.

### 2.5. ***TZ2*** Enhances Chemosensitivity in Lung Cancer Cells

**Figure 4:**
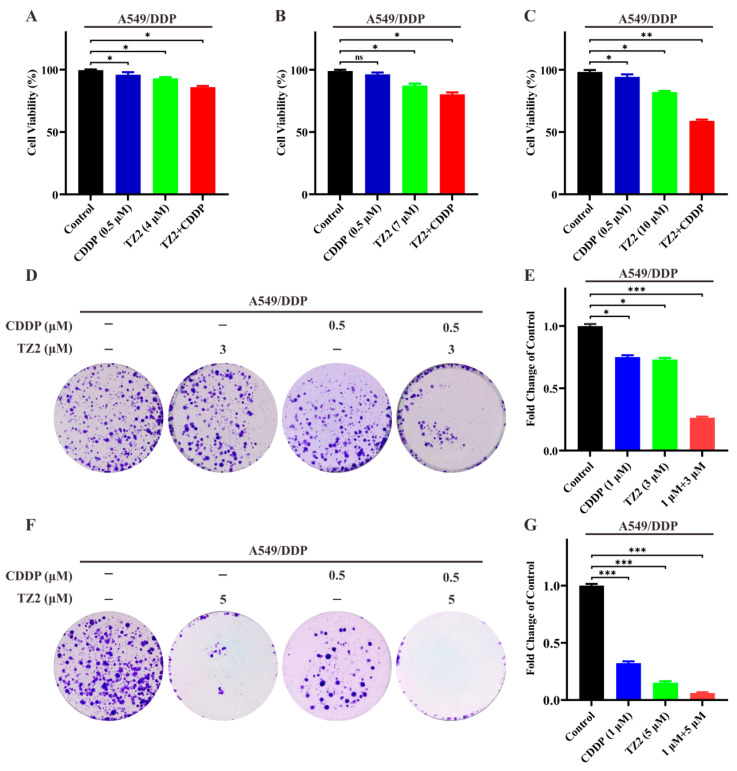
The combination of **TZ2** and cisplatin can reverse chemotherapy resistance in lung cancer cells. (**A**–**C**) Cell viability of A549-CDDP cells treated with the combination of **TZ2** and cisplatin. Cell viability was determined 48 h after treatment, and the proliferation index was calculated as the fold change of cell viability. Columns, means (*n* = 3); bars, standard deviation (* *p* < 0.05, ** *p* < 0.01, *** *p* < 0.001). (**D**–**G**) Colony formation assay in cells treated with **TZ2**, cisplatin, or combination at 14 days. Columns, means (*n* = 3); bars, standard deviation (^ns^ *p* < 0.05, * *p* < 0.05, ** *p* < 0.01, *** *p* < 0.001).

Based on the ability of **TZ2** in regulating the tumor microenvironment, we want to know if **TZ2** could sensitize lung cancer cells to chemotherapy. We next investigated whether **TZ2** could increase the cytotoxicity of cisplatin in A549/DDP. As shown in Figure 4, low-dose cisplatin (0.5 μM) exhibited limited cytotoxicity in cisplatin-resistant cells. However, **TZ2** enhanced the sensitivity of A549/DDP cells to cisplatin in a dose-dependent manner (Figure 4A–C). We next investigated whether **TZ2** had a similar effect on the colony formation of cisplatin-resistant cells by treating them with **TZ2** and cisplatin. Consistent with results in cytotoxicity, the combination had a synergistic effect, which increased the sensitivity of A549/DDP cells to cisplatin for colony formation (Figure 4D–G). These data indicated that **TZ2** can sensitize lung cancer cells to chemotherapy.

## 3. Materials and Methods

### 3.1. Materials and Cell Lines

All reagents and solvents for chemical synthesis were commercially available and used directly without further purification. Maleic anhydride (108-31-6), N-tert-butyloxycarbonyl-1,2-ethylenediamine (57260-73-8), hydrogen chllo-ethyl acetate solution (7647-01-0), potassium 4-sulfo-1, 8-naphthalene anhydride (71501-16-1), N-(2-aminoethyl) morpholine (2038-03-1), N,N-diisopropylethylamine (7087-68-5) were purchased from Energy Chemical. Sodium acetate (127-09-3), acetic anhydride (108-24-7), sodium bicarbonate (144-55-8), sodium chloride (7647-14-5), dimethyl sulfoxide (67-68-5), N,N-dimethylformamide (68-12-2), toluene (108-88-3), ethanol (64-17-5), ethyl acetate (141-78-6), and dichloromethane (75-09-2) were purchased from Xilong Technology (Shantou, China). PBS was purchased from Shenzhen Mohong Technology Co., Ltd. (Shenzhen, China). ^1^H and ^13^C spectra were recorded using a Bruker DRX-400MHz with tetramethylsilane as an internal standard (Billerica, MA, USA). High-resolution mass spectra were recorded in a Thermo Exactive Orbitrap mass spectrometer using electrospray ionization (ESI) (Waltham, MA, USA). The purity of the synthetic compounds was higher than 95% as determined by HPLC. The characterization data of **TZ2** were summarized in Figures S1–S4.

Anti-Cyclin B1 (AF1606) and anti-CDK1 (AF1516) antibodies were obtained from Beyotime (Shanghai, China). Anti-GAPDH (M1211-1) antibody was purchased from HUABIO (Hangzhou, China). Anti-CDK2 (ab32147) antibody was obtained from EPITMICS (Burlingame, CA, USA). Anti-Cyclin A2 (GTX103042) antibody was purchased from GeneTex (Irvine, CA, USA). Anti-rabbit and anti-mouse secondary antibodies were obtained from ZSGB-BIO (Shanghai, China). 1 × PBS (pH 7.2–7.4, 0.01 M, cell culture, P1020), crystal violet (C8470), and NP40 (N8030) were purchased from Solarbio (Beijing, China). A549/DDP cell line (human lung cancer with acquired cisplatin resistance) was obtained from Pricella. NCI-H460 (human lung cancer cells), A549 (human lung cancer cells), and HepG2 (human liver cancer cells) cell lines were provided by the Stem Cell Bank, Chinese Academy of Sciences. The NCI-H460, A549, A549/DDP, and WI-38 cell lines were cultured in RPMI 1640 medium (GIBCO-BRL, Grand Island, NY, USA) supplemented with 10% fetal calf serum (FCS). The HepG2 was cultured in DMEM medium (GIBCO-BRL, Grand Island, NY, USA) containing 10% fetal calf serum (FCS).

### 3.2. General Procedure for the Synthesis of Compounds ***TZ2***

Maleic anhydride (490.3 mg, 5 mmol) and N-Boc-1,2-ethylenediamine (961.32 mg, 6 mmol) in dichloromethane, stirring at room temperature. The product reacted with sodium acetate (451.2 mg, 5.5 mmol) in acetic anhydride at 65 °C, followed by dichloromethane extraction, concentration under reduced pressure, and column chromatography to yield Compound 1 as a white solid. Compound 1 was treated with ethyl acetate containing HCl (5.5 mmol) at room temperature, and suction filtration to afford Compound 2. Separately, 4-sulfo-1,8-naphthalic anhydride potassium salt (632.66 mg, 0.2 mmol) was dissolved in anhydrous ethanol with N-(2-aminoethyl)morpholine (520.76 mg, 0.4 mmol), and the mixture was refluxed under nitrogen protection. After cooling in an ice bath, filtered and dried to obtain Compound 3. Compound 3 was dissolved in thionyl chloride (20 mL) with catalytic DMF, refluxed, and concentrated via azeotropic distillation with toluene to yield Compound 4 as a white solid. Finally, Compound 4 was dissolved in anhydrous ethanol, treated with N,N-diisopropylethylamine (646.25 mg, 5 mmol) and Compound 2 (369.648 mg, 2.1 mmol), stirred at room temperature, extracted and purified by column chromatography to afford **TZ2** as white solids in 70% yield.

^1^H NMR (400 MHz, Chloroform-d) δ 8.96 (dd, *J* = 8.6, 1.2 Hz, ^1^H), 8.67 (dd, *J* = 7.3, 1.1 Hz, ^1^H), 8.62 (d, *J* = 7.7 Hz, ^1^H), 8.39 (d, *J* = 7.7 Hz, ^1^H), 7.91 (dd, *J* = 8.7, 7.3 Hz, ^1^H), 6.52 (s, ^2^H), 5.58 (t, *J* = 6.1 Hz, ^1^H), 4.37 (t, *J* = 6.4 Hz, ^2^H), 3.70 (d, *J* = 7.1 Hz, ^4^H), 3.63–3.59 (m, ^2^H), 3.26 (q, *J* = 5.8 Hz, ^2^H), 2.77 (s, ^2^H), 2.67 (d, *J* = 13.3 Hz, ^4^H).(^13^C NMR) ^13^C NMR (100 MHz, Chloroform-d) δ 171.00, 163.95, 163.34, 140.77, 134.36, 132.28, 131.32, 130.12, 129.75, 129.54, 129.43, 127.22, 127.20, 123.70, 67.39, 56.48, 54.24, 42.54, 37.87, 37.71, 1.48. HRMS (ESI) *m*/*z* calcd for C_24_H_25_N_4_O_7_S ([M + H]^+^) 513.1438, found 513.1451. HPLC: 96.58%, *t*_R_ = 6.824 min.

### 3.3. Method for Selective Reaction of ***TZ2*** with GSH

Fluorescence-selective reactions were captured using a ZF-5 handheld UV analyzer. Fluorescence spectra were acquired using a SHIMADZU RF-5301 fluorescence spectrophotometer. A stock solution of **TZ2** (2 mM) was prepared in DMSO, and stock solutions of the amino acids (Gly, Ala, Cys, Ser, Arg, Phe, Pro) and GSH (each 10 mM) were dissolved in distilled water. For selectivity studies, a working solution of **TZ2** (5 μM) was prepared by mixing aliquots of the **TZ2** stock solution (2 mM) and amino acid or GSH stock solutions (0.1 mM) in 1 × PBS buffer/DMSO (19:1, *v*/*v*). A solution containing **TZ2** (5 μM) and amino acid solutions or GSH (10 mM final concentration) in 1 × PBS buffer was mixed for 5 h. Under dark conditions, the sample was exposed to the UV analyzer, and pictures were taken with a camera. Then, the sample was added to a quartz cuvette. After the quartz cuvette was placed in the designated position, the emission spectra were recorded from 365 to 700 nm upon excitation at 502 nm, with excitation and emission slit widths both set to 5 nm.

### 3.4. Method for the Reaction Time of ***TZ2*** with GSH

In the fluorescence reaction time test, a solution containing **TZ2** (5 μM) and GSH (10 mM) was placed in 1 × PBS buffer and incubated for different times (0, 30, 60, 150, 300 min). Then, the samples were added to a quartz cuvette. The quartz cuvette was placed behind a fluorescence spectrophotometer, and the emission spectra were recorded from 365 to 700 nm under 502 nm excitation, with both excitation and emission slit widths set to 5 nm.

### 3.5. Determination of the Reaction Products Between ***TZ2*** and GSH in Different pH

A high-performance liquid chromatograph (HPLC) from Agilent Technologies was used for the separation and analysis of analytes. Fluorescence spectra were acquired using a SHIMADZU RF-5301 fluorescence spectrophotometer. For the pH test, solutions containing **TZ2** (5 μM) and GSH (10 mM) were incubated in different pH buffer solutions for 5 h. The samples were then transferred to liquid chromatography vials, placed in the designated positions for liquid phase mass spectrometry, and analyzed using a 5-min gradient elution (10% to 100% methanol–water). After this, the samples were loaded into quartz cuvettes. Once the cuvettes were positioned in the fluorescence spectrometer, emission spectra were recorded from 365 nm to 700 nm under 502 nm excitation, with both excitation and emission slit widths set to 5 nm.

### 3.6. IC_50_ Value Determination

Cells were seeded in a 96-well plate and treated with various concentrations of **TZ2** (1.25, 2.5, 5, 10, and 20 μM) for 48 h. Each concentration was tested five times, and a blank control was included. Then, 10 μL MTT (5 mg/mL) was added to each well, and the cells were incubated for 5 h. After treatment, the culture medium was discarded, 100 μL DMSO was added, the plate was shaken, and the absorbance was measured at 490 nm. Finally, the IC_50_ value was calculated using the SPSS 20 software by taking the average of the three replicate experiments.

### 3.7. Cell Viability Assay

Cells were seeded into 96-well plates and treated with CDDP (0.5 μM) and **TZ2** (4, 7, 10 μM) for 48 h. Each concentration was tested in quintuplicate, with a blank control group. Subsequently, 10 μL of MTT (5 mg/mL) was added to each well, and the cells were incubated for 5 h. After treatment, the culture medium was discarded, 100 μL of DMSO was added, the plate was shaken, and the absorbance at 490 nm was measured. The cell survival rate was calculated as the average of three replicate experiments.

### 3.8. Fluorescence Imaging

Cells were inoculated in six-well plates, after the drug intervention, cleaned twice with PBS, and then 1 mL PBS was added for intracellular fluorescence imaging detection immediately through the multifunctional imaging system, and the GFP channel was selected.

### 3.9. Colony Formation Assay

Cells were seeded into six-well plates at a density of 500 cells per well. 24 h later, cells were treated with CDDP (0.5 μM) and **TZ2** (0.5, 1, or 3 μM) for 14 d, and the culture medium was replaced with fresh culture medium every three days. After the cells formed visible colonies, they were fixed with fixative and stained with 0.1% crystal violet solution for 15 min. After staining, the cells were washed with PBS to remove the residual dye, dried naturally, and then photographed for imaging.

### 3.10. Western Blotting

A549 and HepG2 cells were seeded into six-well plates. After treating the cells with **TZ2** (0, 3, 6, 9 μM) for 48 h, the cells were lysed. Then, the cell extracts were separated on SDS-polyacrylamide gels, transferred to nitrocellulose membranes, and incubated with the indicated primary and secondary antibodies. The images were developed using an enhanced chemiluminescence imaging analyzer (Tanon-5200, Shanghai, China).

### 3.11. Statistical Analysis

All data are shown as mean ± standard deviation (SD) using two-tailed Student’s *t* tests and one-way ANOVA with Bonferroni multiple comparison post-test—*p* values less than 0.05 were considered significant differences.

## 4. Conclusions

A novel tumor microenvironment-responsive agent, **TZ2**, featuring a naphthylimide-based molecular framework, was successfully developed with glutathione (GSH)-activated functionality. This diagnostic–therapeutic agent demonstrates pH-dependent fluorescence response (502 nm emission) within the physiological pH range (6–11), exhibiting initial fluorescence enhancement followed by gradual attenuation. Specificity analysis revealed selective fluorescence activation exclusively upon GSH interaction, with no significant response observed with other biological thiols. Cellular internalization studies confirmed **TZ2**′s ability to engage with endogenous GSH, generating fluorescent metabolites within tumor cells. **TZ2** exhibited antiproliferative effects against multiple cancer cell lines (A549, NCI-H460, HepG2). **TZ2** exhibited dual functionality, both as a tumor microenvironment indicator and a therapeutic agent. Significantly, combination therapy with cisplatin (CDDP) revealed **TZ2′**s capacity to sensitize chemoresistance in lung cancer models, suggesting potential for synergistic treatment strategies.

## Figures and Tables

**Table 1:** Anticancer activities of **TZ2** in several cancer cell lines.

Comp.	IC_50_ (μM) *^a^*			
	NCI-H460	A549	HepG2	WI-38
**TZ2**	>20	14.51 ± 0.76	>20	>20

*^a^* Data shown are means with SD of three independent experiments.

## Data Availability

The experimental data used to support the results of this study are available in the article and in the Supplementary Materials.

## Supplementary Materials

The following supporting information can be downloaded at: https://www.mdpi.com/article/10.3390/molecules30153081/s1. 1. Chemical characterization of TZ2, Figures S1–S4; 2. HRMS (ESI) spectrum of TZ2-1 and TZ2-2, Figures S5 and S6; 3. HRMS (ESI) spectrum of TZ2 and GSH at different pH conditions, Figures S7–S17; 4. Full unprocessed fluorescence imaging; 5. Full and uncropped western blots.
