# Supplementary material for: A pH-Sensitive Glutathione Responsive Small-Molecule Probe TZ2 Sensitizes Lung Cancer Cells to Chemotherapy by Targeting Tumor Microenvironment
Source: Molecules. 2025 Jul 23;30(15):3081. doi: 10.3390/molecules30153081 (PMC12348317; doi:10.3390/molecules30153081)
Supplement: Supplementary file 1 [file molecules-30-03081-s001.zip › molecules-3700013-supplementary.pdf]

Supporting Information

A pH-Sensitive Glutathione Responsive Small-Molecule Probe TZ2 Sensitizes Lung Cancer Cells to Chemotherapy by Targeting Tumor Microenvironment

Changle Zhong †, Minghan Lu †, Guanhao Pan †, Xintong You, Yan Peng, Shulan Zeng * and Guohai Zhang *

Key Laboratory for Chemistry and Molecular Engineering of Medicinal Resources (Ministry of Education of China), Guangxi Key Laboratory of Chemistry and Molecular Engineering of Medicinal Resources, School of Chemistry and Pharmaceutical Sciences, Guangxi Normal University, Guilin 541004, China; 2022010365@gxnu.edu.cn (C.Z.); 2022010356@gxnu.edu.cn (M.L.); 3033542093@gxnu.edu.cn (G.P.); 18851876191@163.com (X.Y.); pegnyan630@gxnu.edu.cn (Y.P.)

* Correspondence: zengsl@gxnu.edu.cn (S.Z.); zgh1207@gxnu.edu.cn (G.Z.)

† These authors contributed equally to this work.

Key Words: tumor microenvironment; GSH depletion; pH response; drug resistance

This PDF file includes：

1. Chemical characterization of TZ2, Fig. S1 to S4
2. HRMS（ESI）spectrum of TZ2-1 and TZ2-2, Fig. S5 to S6
3. HRMS（ESI）spectrum of TZ2 and GSH at different pH conditions, Fig. S7 to S17
4. Full unprocessed fluorescence imaging
5. Full and uncropped western blots

Chemical characterization of TZ2

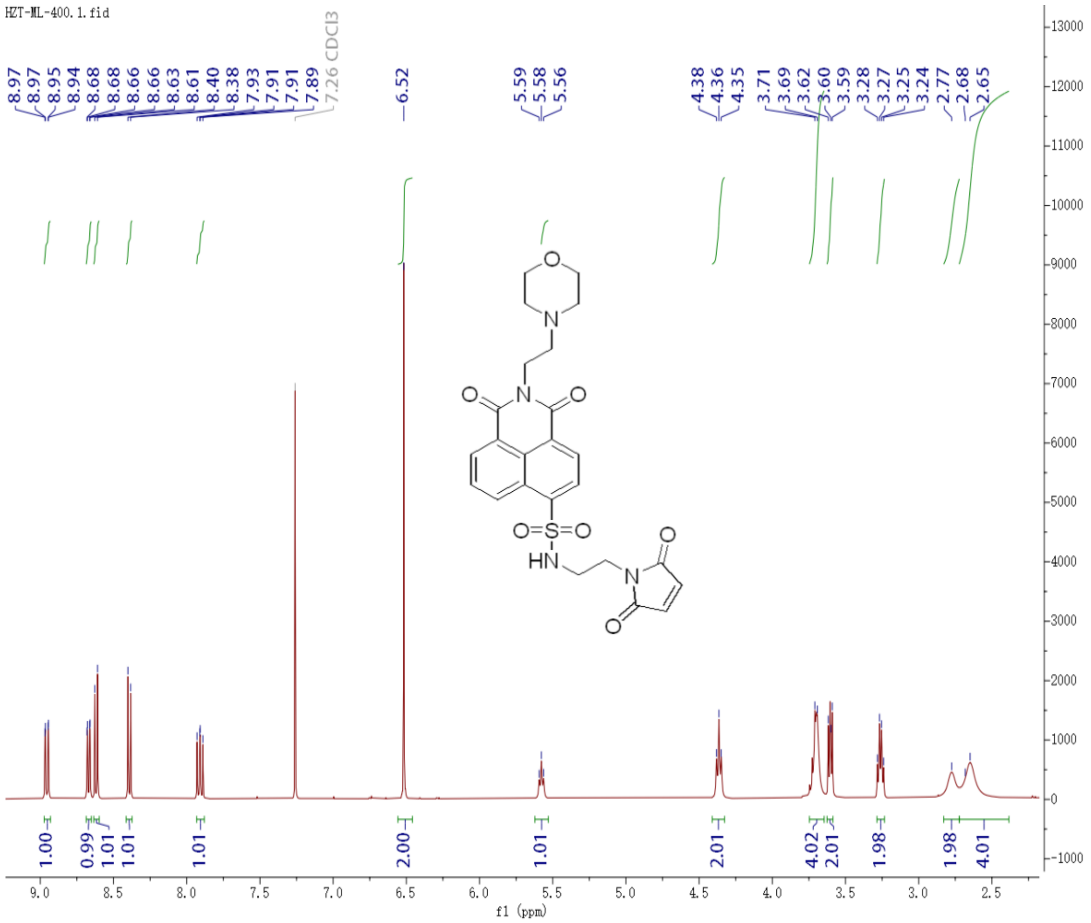

Supplementary Figure S1.^{1}H NMR spectrum of **TZ2** in Chloroform-d

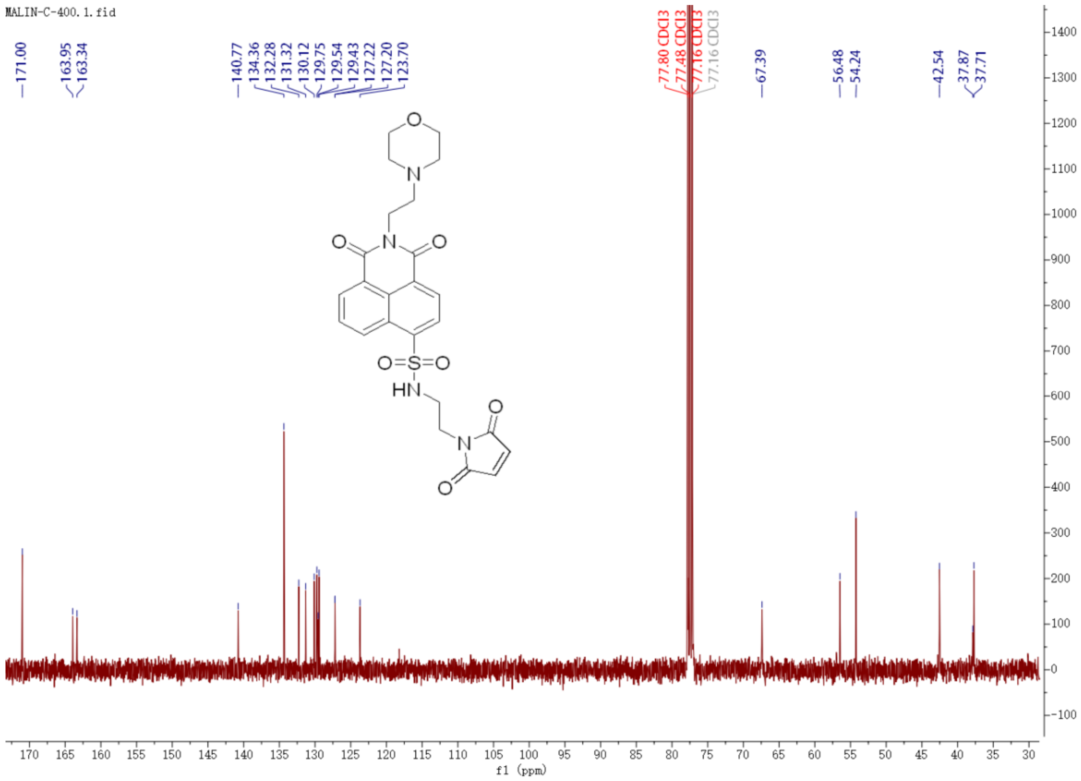

Supplementary Figure S2. ^{13}C NMR spectrum of **TZ2** in Chloroform-d

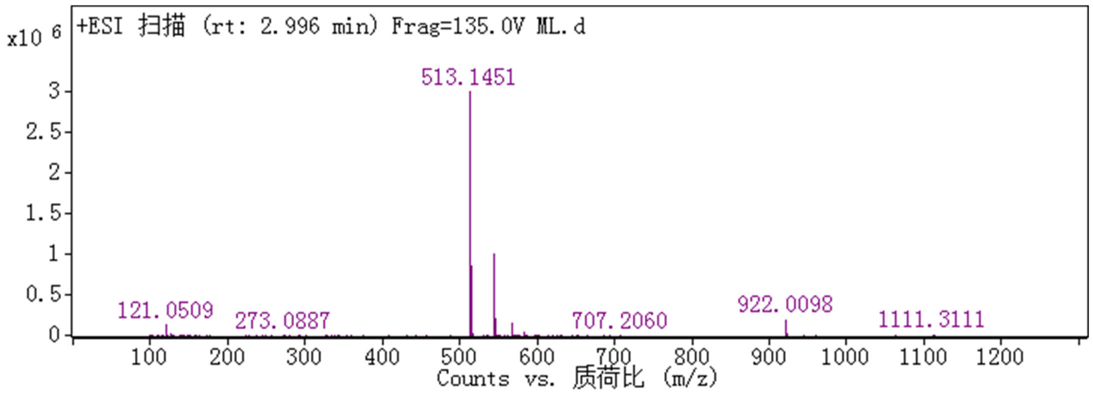

Supplementary Figure S3. HRMS（ESI）spectrum of **TZ2**

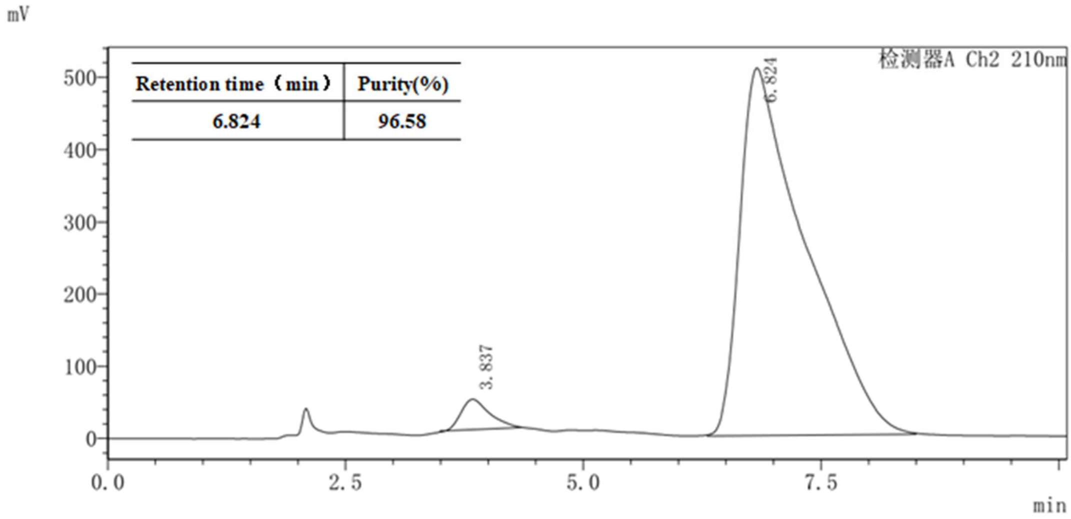

Supplementary Figure S4. HPLC of **TZ2**（mobile phase: 35% methyl alcohol-water）

HRMS（ESI）spectrum of TZ2-1 and TZ2-2

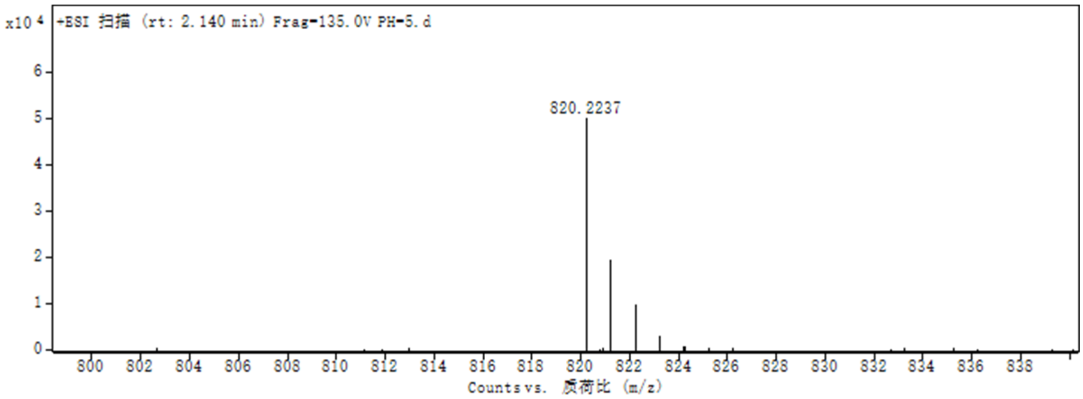

Supplementary Figure S5. HRMS（ESI）spectrum of **TZ2-1**

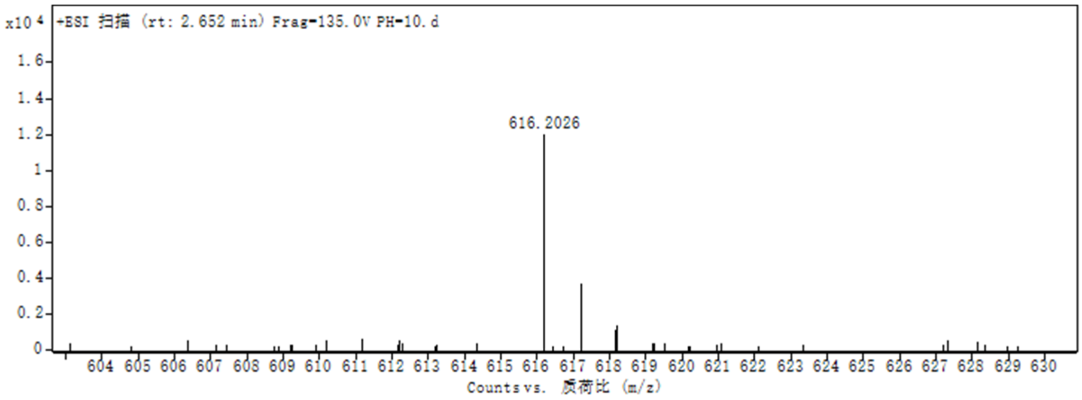

Supplementary Figure S6. HRMS（ESI）spectrum of **TZ2-2**

HRMS（ESI）spectrum of TZ2 and GSH at different pH conditions

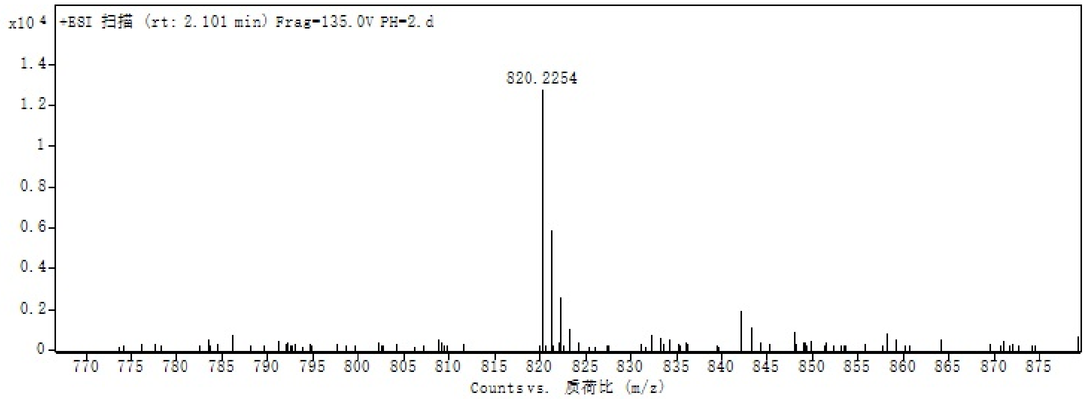

Supplementary Figure S7. HRMS（ESI）spectrum of **TZ2-1** formed by the reaction of **TZ2** with GSH at pH 2.

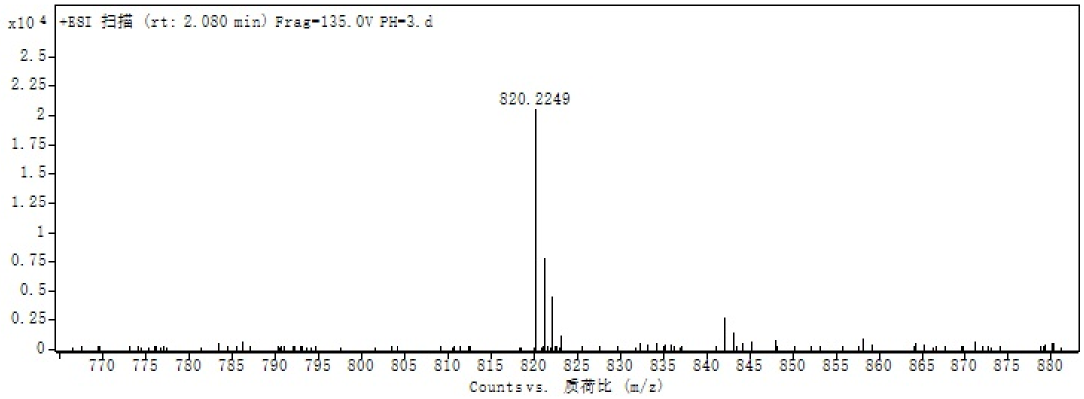

Supplementary Figure S8. HRMS（ESI）spectrum of **TZ2-1** formed by the reaction of **TZ2** with GSH at pH 3.

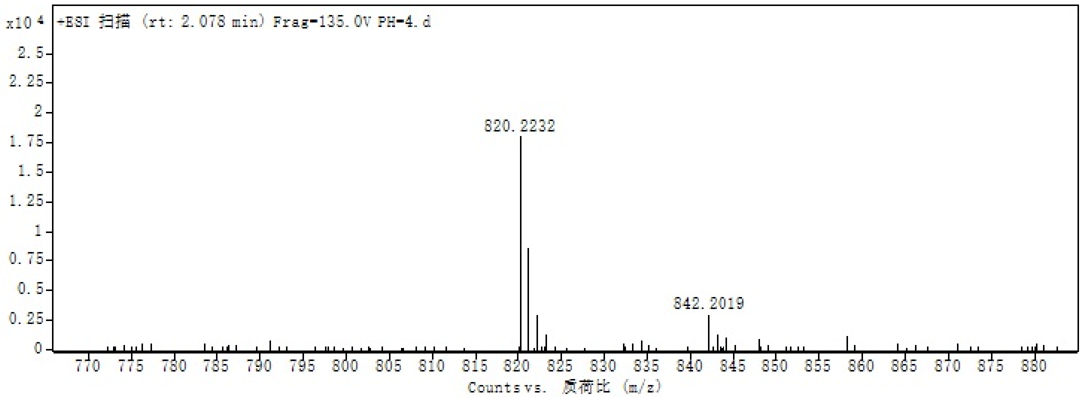

Supplementary Figure S9. HRMS（ESI）spectrum of **TZ2-1** formed by the reaction of **TZ2** with GSH at pH 4.

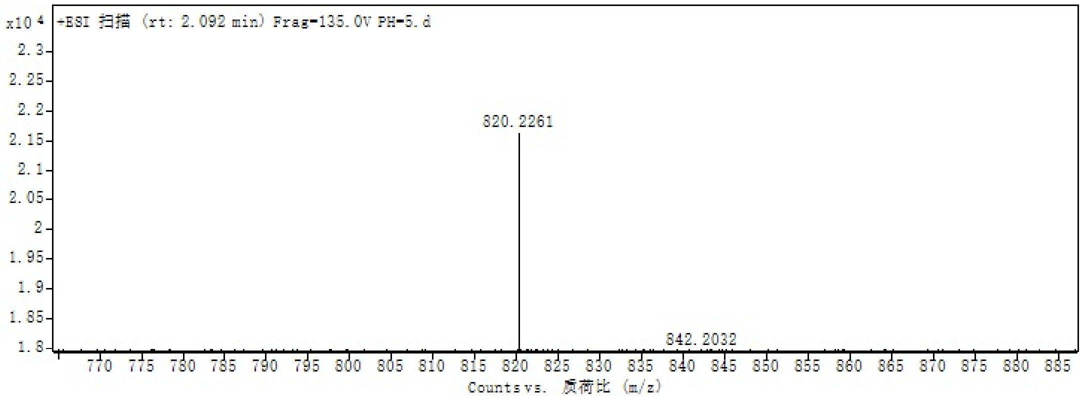

Supplementary Figure S10. HRMS (ESI) spectrum of **TZ2-1** formed by the reaction of **TZ2** with GSH at pH 5.

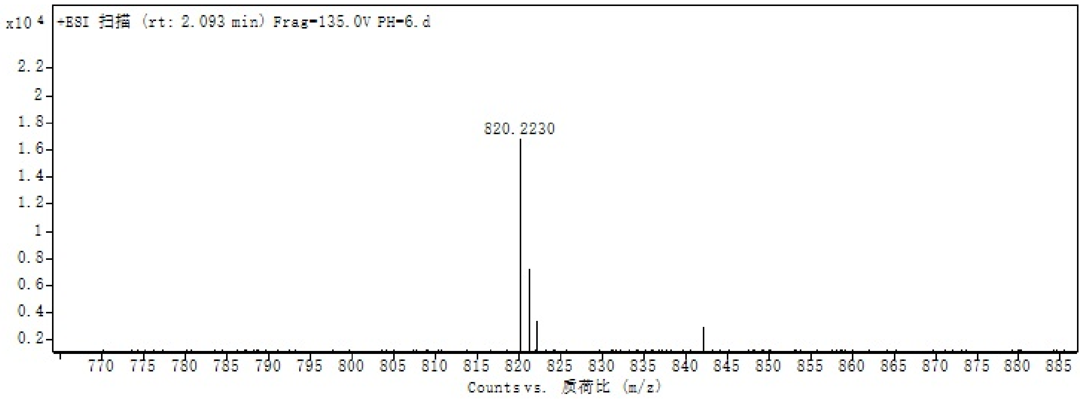

Supplementary Figure S11. HRMS (ESI) spectrum of **TZ2-1** formed by the reaction of **TZ2** with GSH at pH 6.

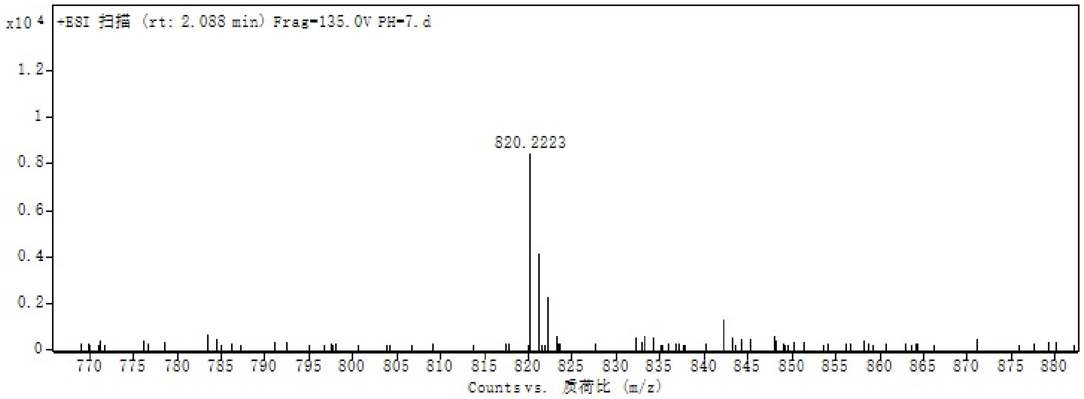

Supplementary Figure S12. HRMS (ESI) spectrum of **TZ2-1** formed by the reaction of **TZ2** with GSH at pH 7.

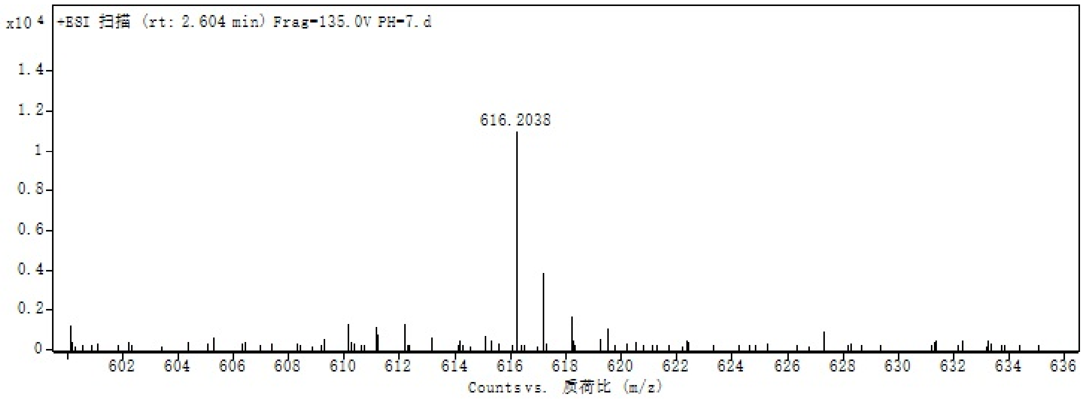

Supplementary Figure S13. HRMS (ESI) spectrum of **TZ2-2** formed by the reaction of **TZ2** with GSH at pH 7

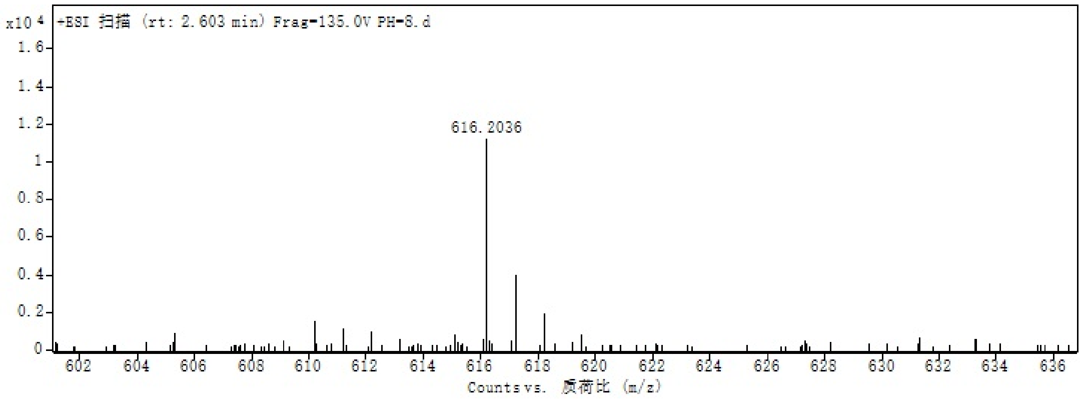

Supplementary Figure S14. HRMS (ESI) spectrum of **TZ2-2** formed by the reaction of **TZ2** with GSH at pH 8

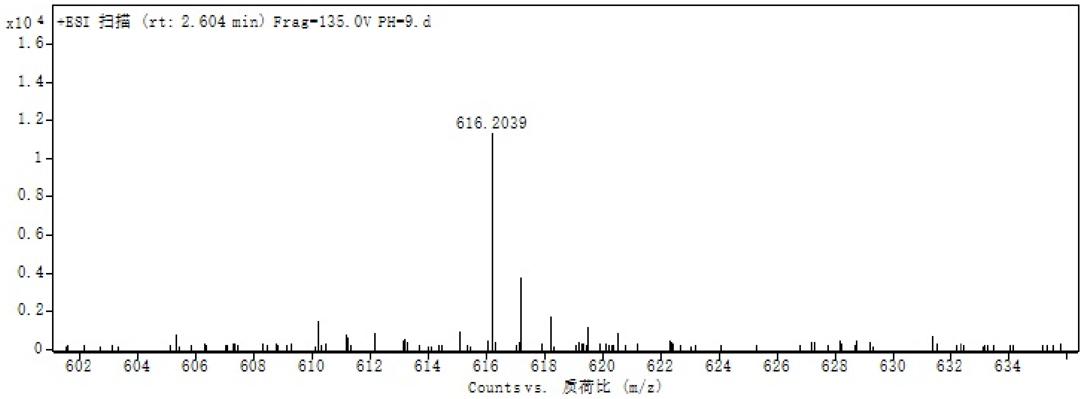

Supplementary Figure S15. HRMS (ESI) spectrum of **TZ2-2** formed by the reaction of **TZ2** with GSH at pH 9

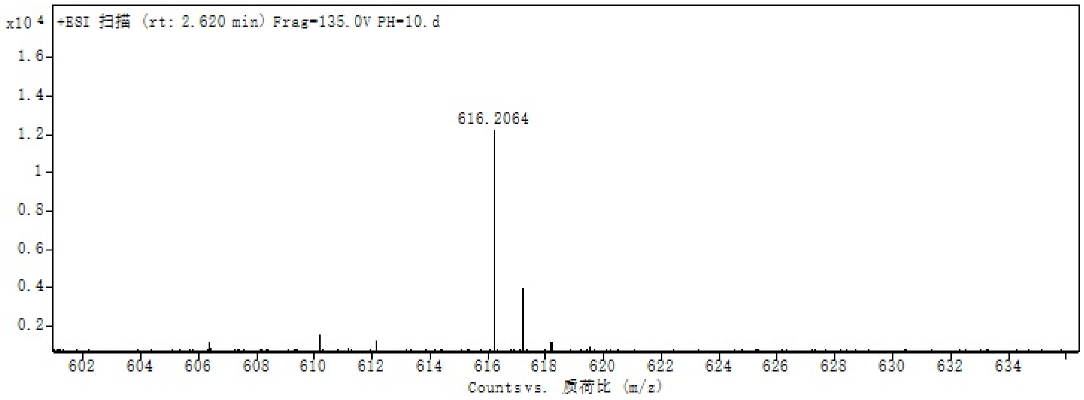

Supplementary Figure S16. HRMS (ESI) spectrum of **TZ2-2** formed by the reaction of **TZ2** with GSH at pH 10

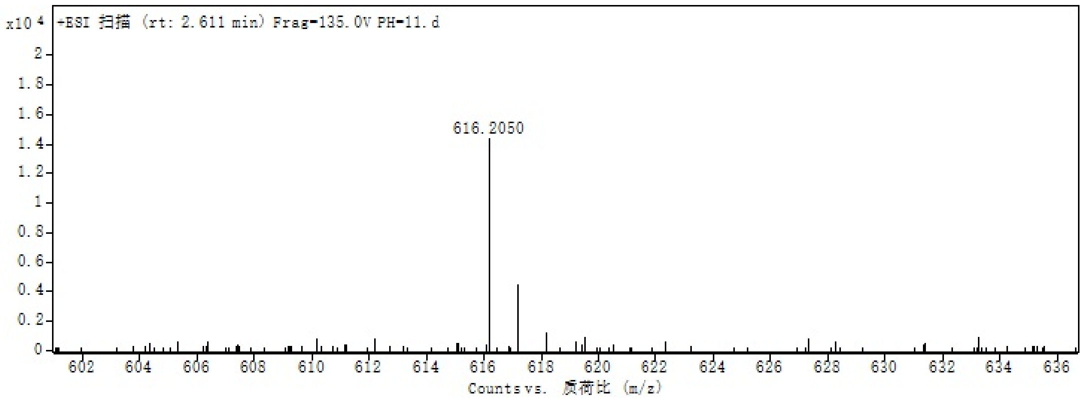

Supplementary Figure S17. HRMS (ESI) spectrum of **TZ2-2** formed by the reaction of **TZ2** with GSH at pH 11

Full unprocessed fluorescence imaging

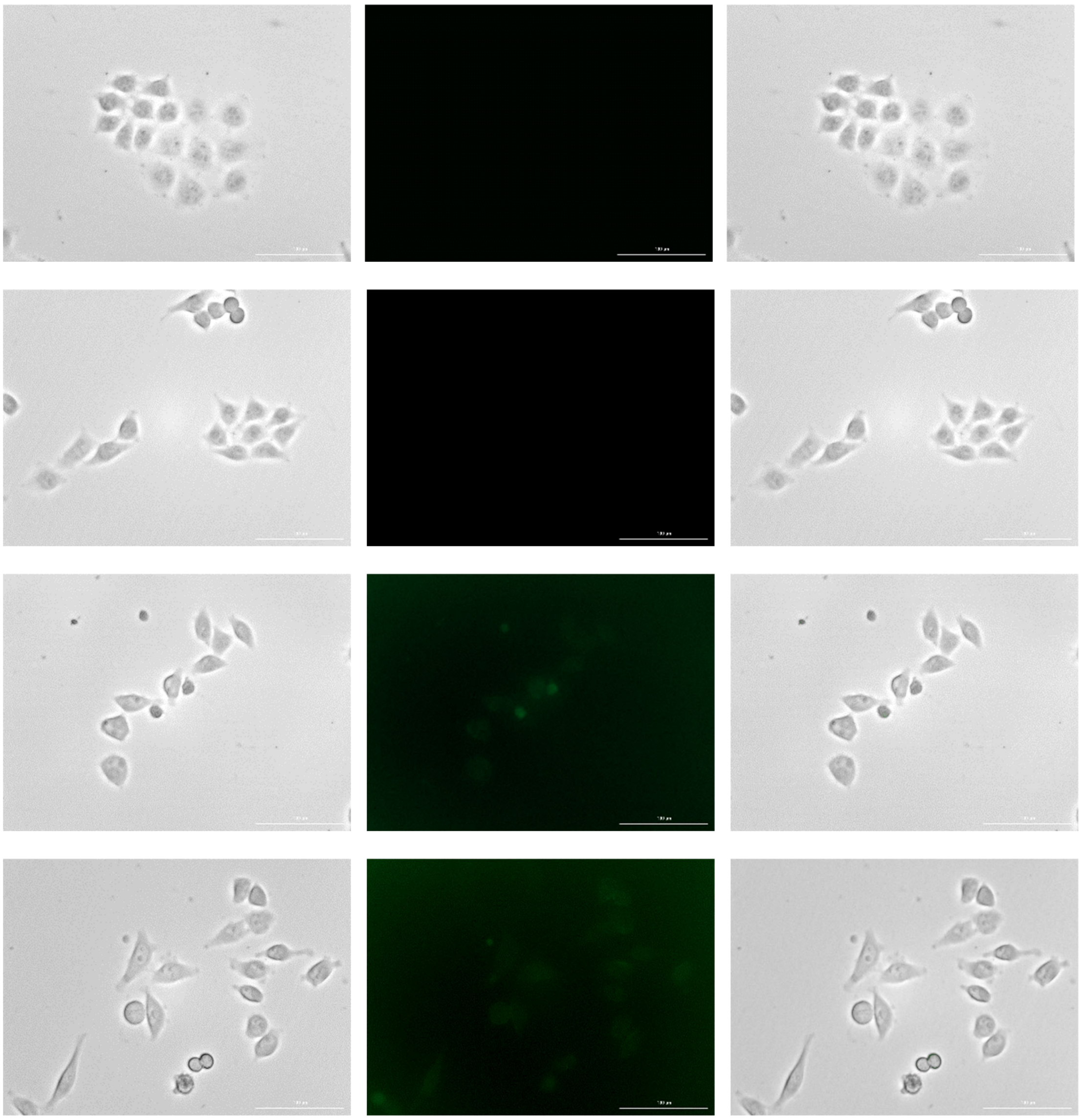

Fluorescence imaging raw data in Figure 2A

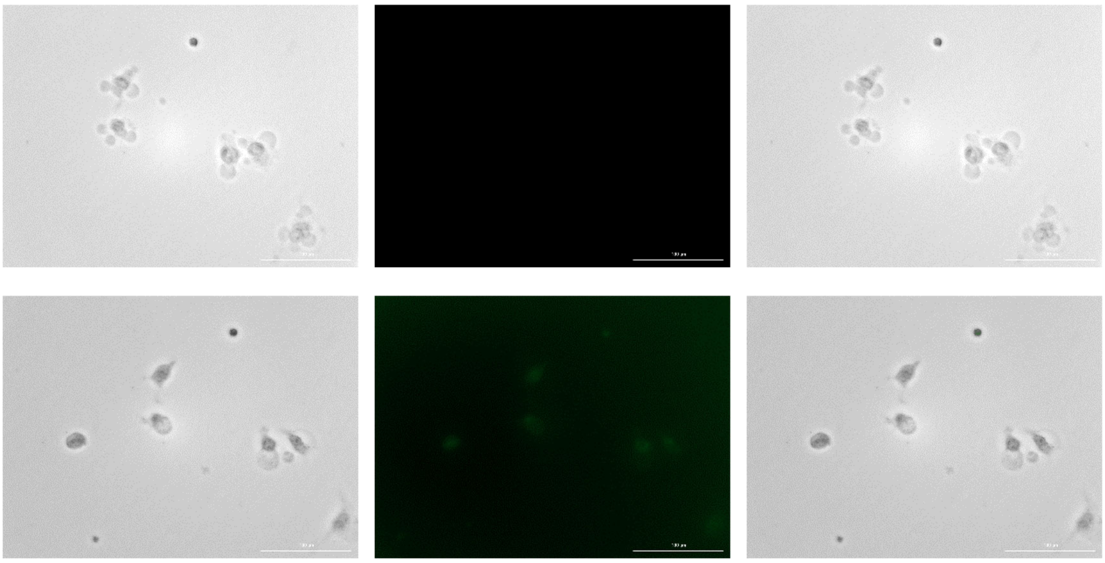

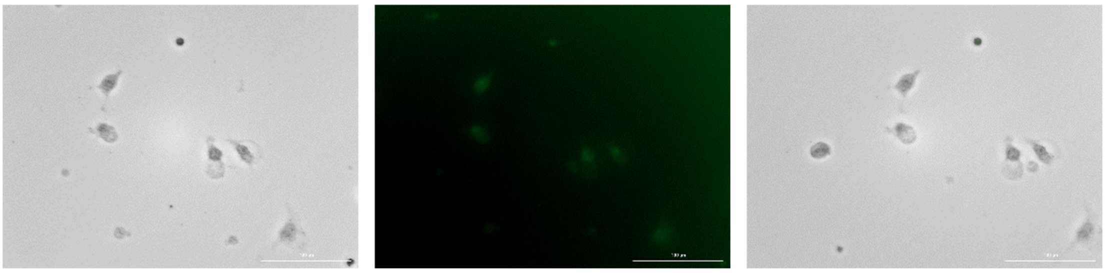

Fluorescence imaging raw data in Figure 2B

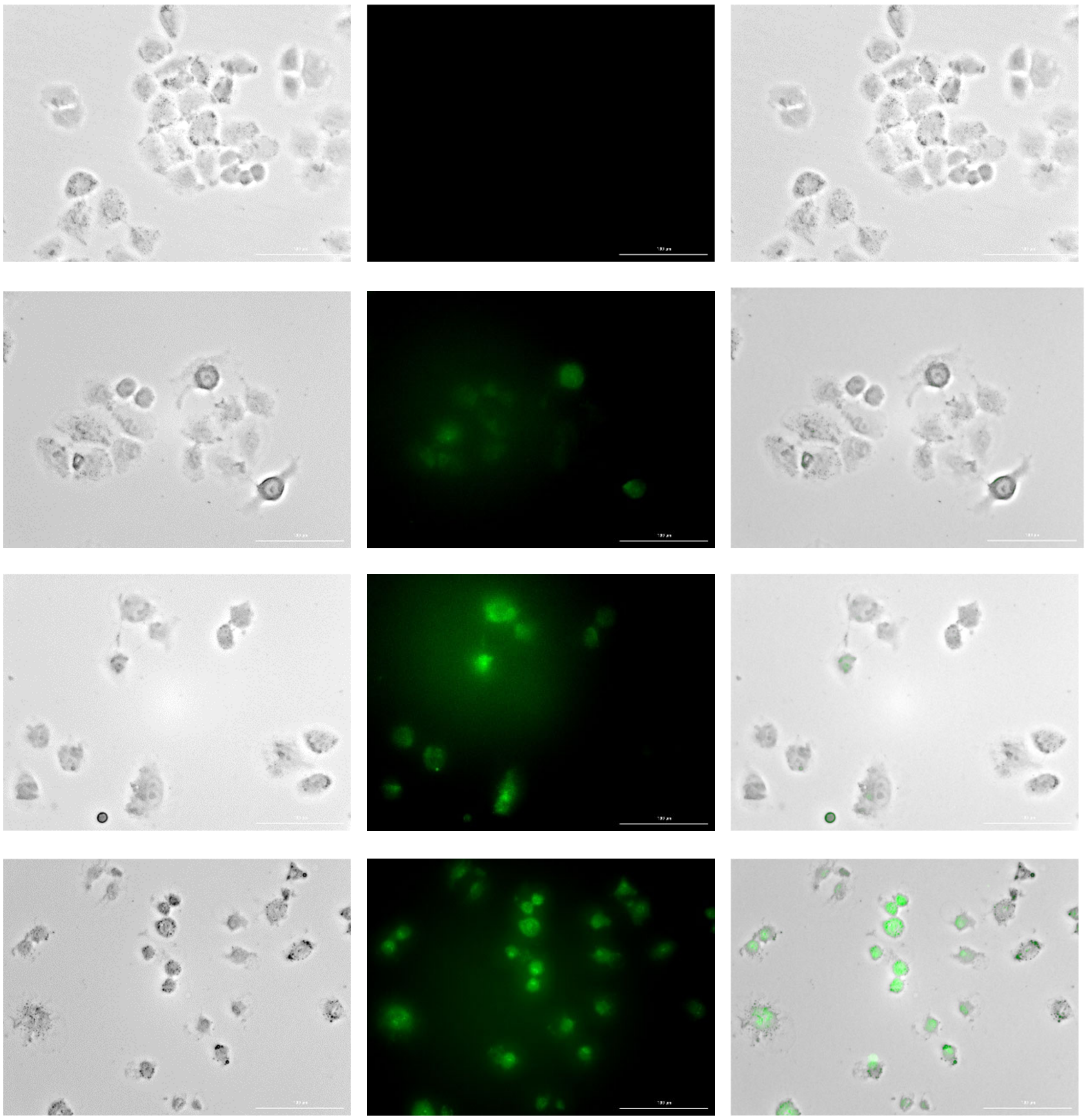

Fluorescence imaging raw data in Figure 2C

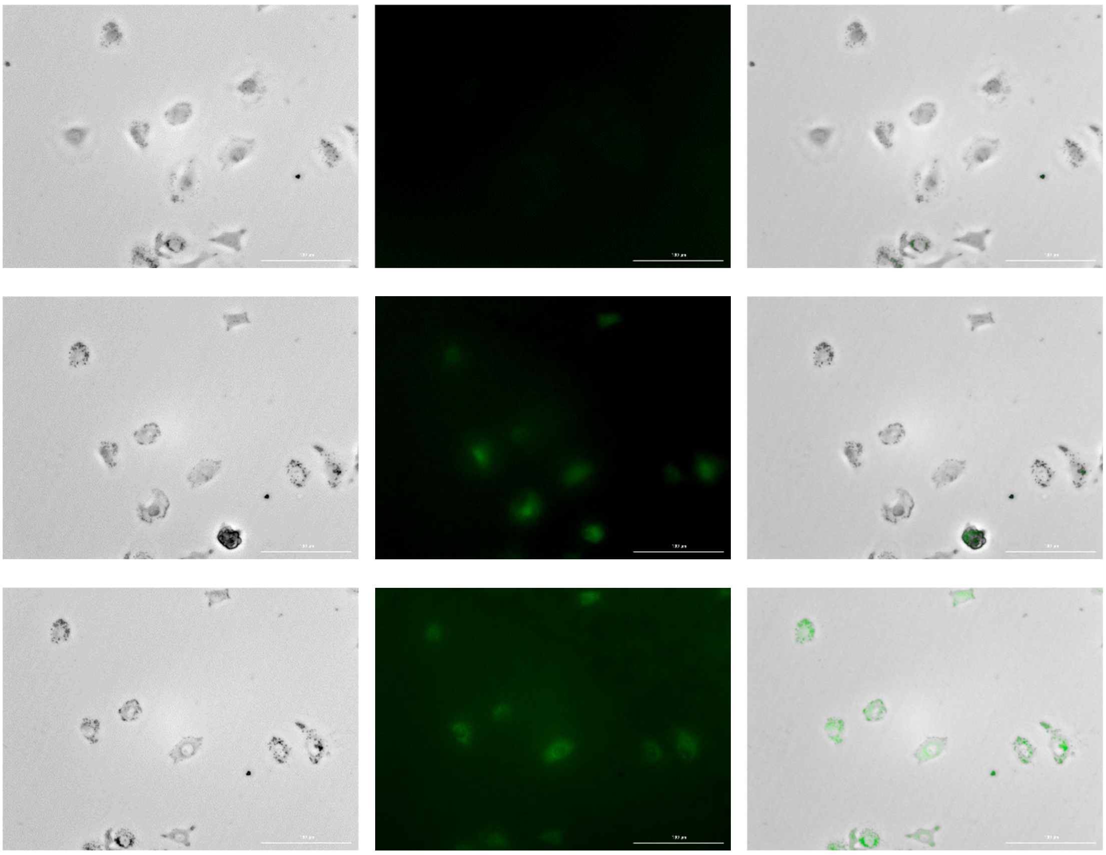

Fluorescence imaging raw data in Figure 2D

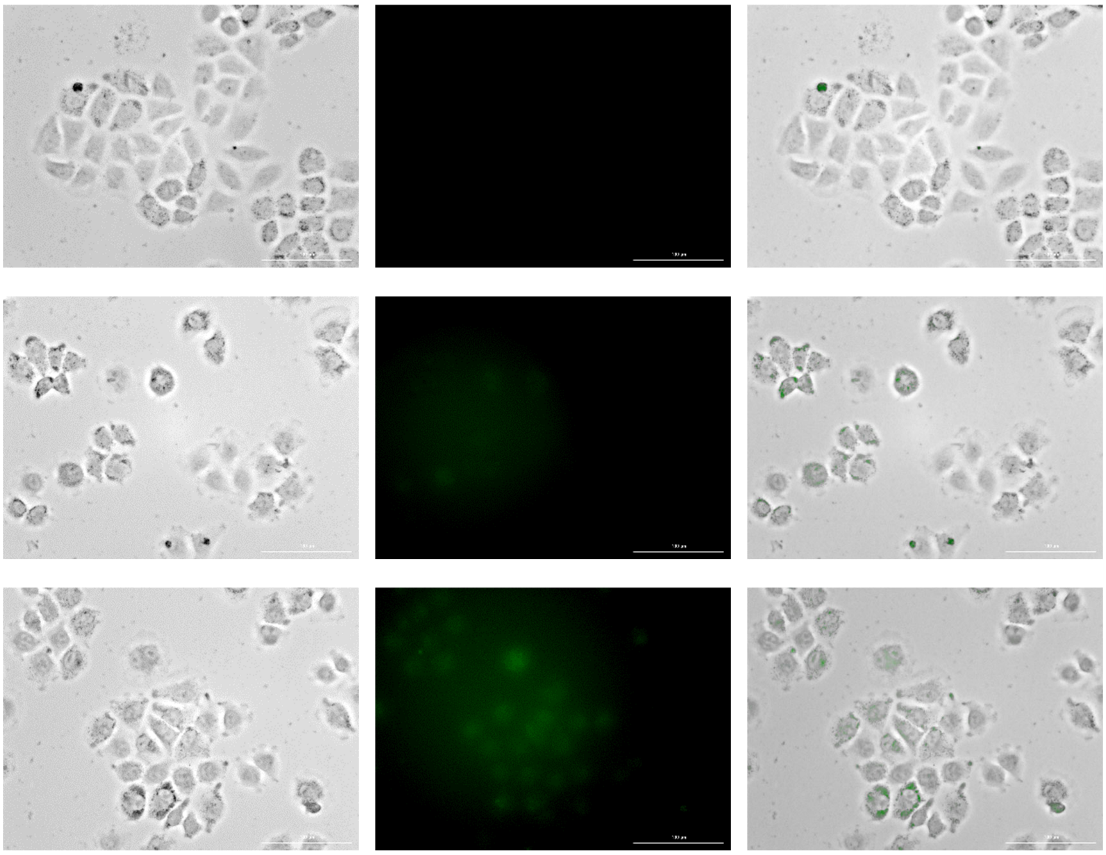

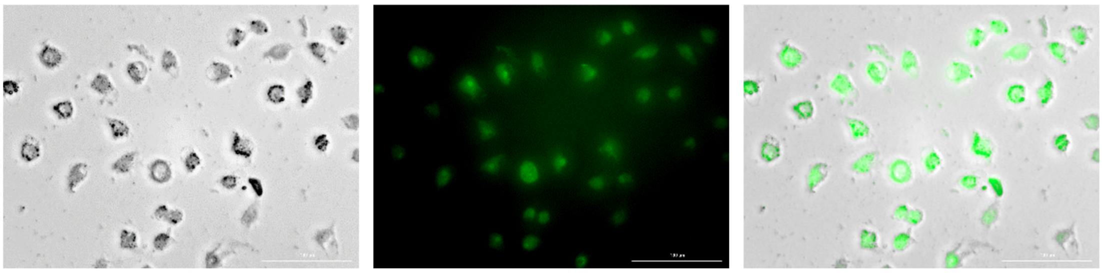

Fluorescence imaging raw data in Figure 2E

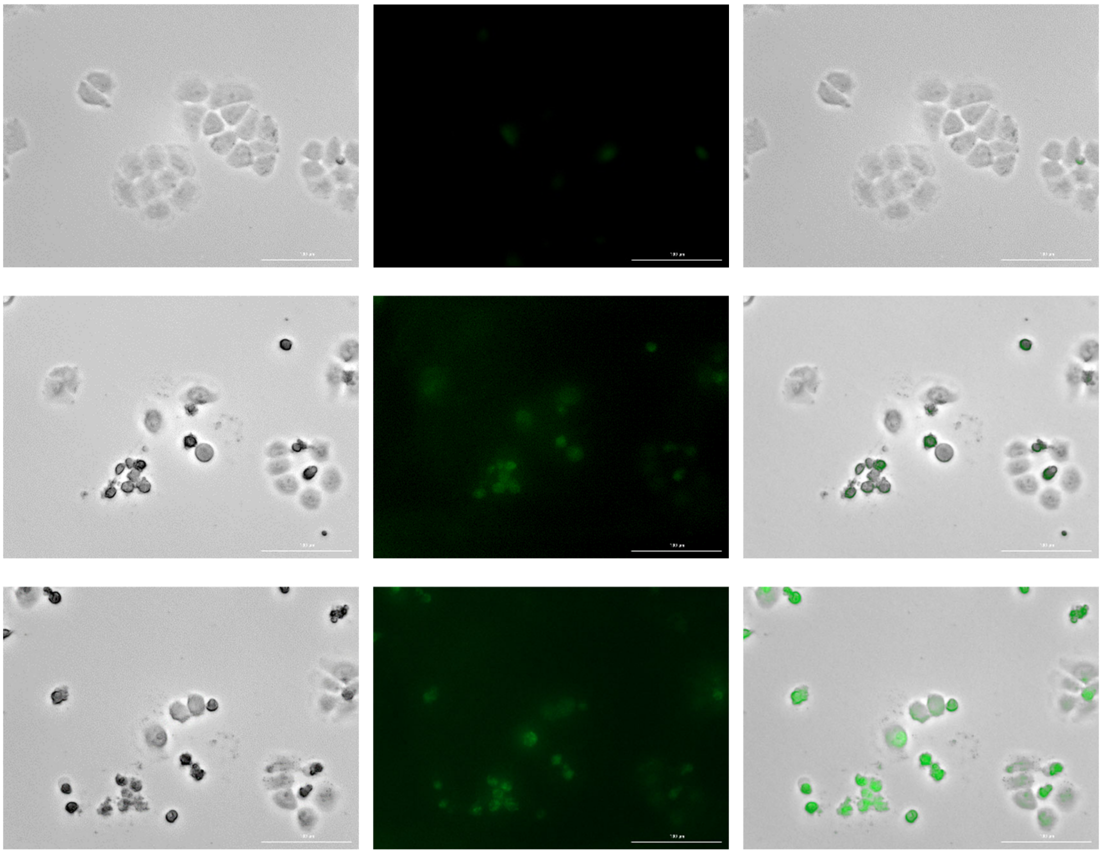

Fluorescence imaging raw data in Figure 2F

Full and uncropped western blots

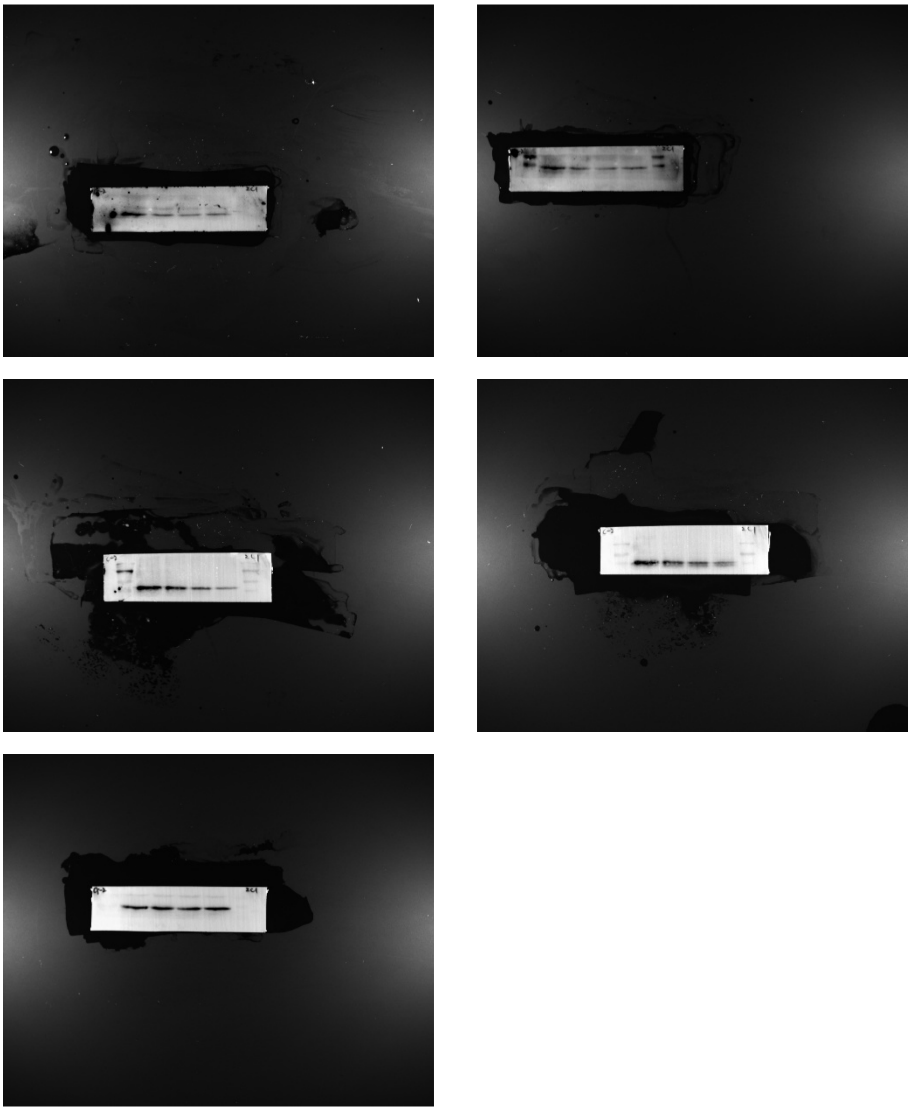

Western blot raw data in Fig. 3G

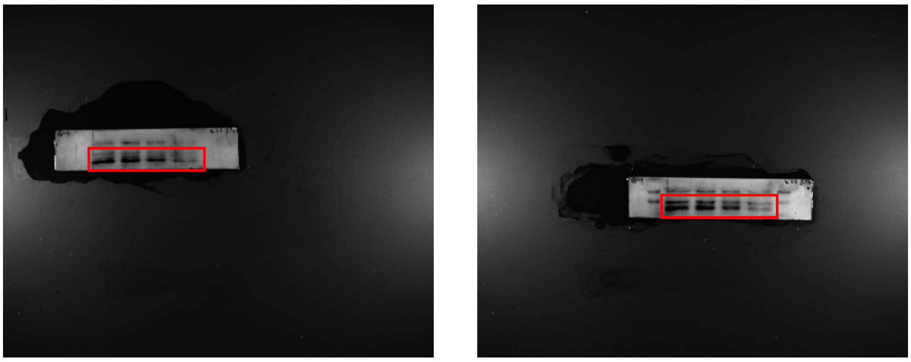

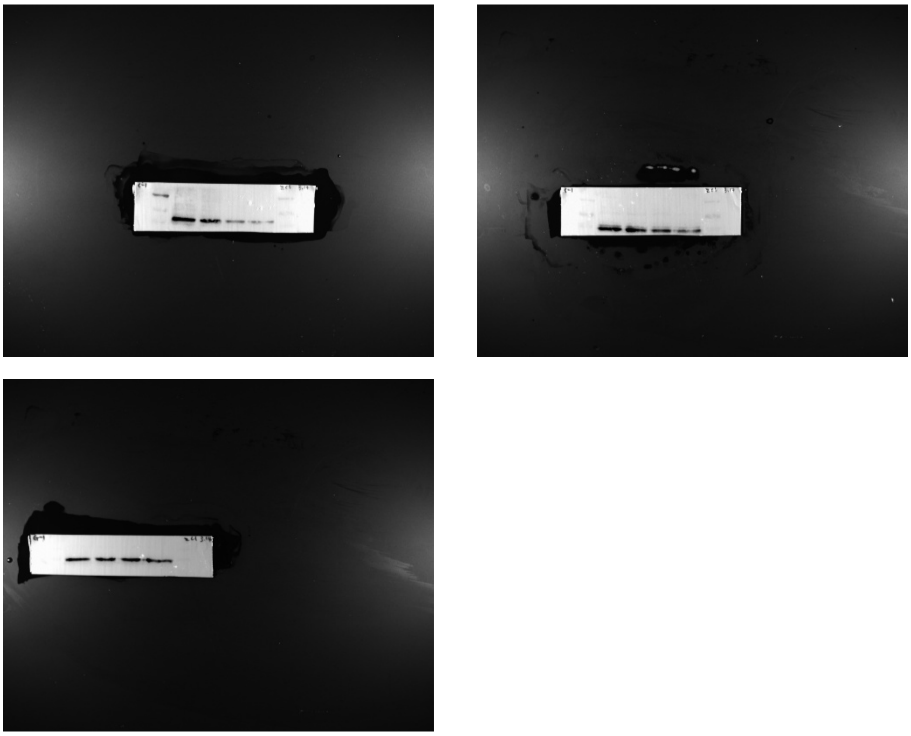

Western blot raw data in Fig. 3I
